# Assessment of Stromal Invasion for Correct Histological Diagnosis of Early Hepatocellular Carcinoma

**DOI:** 10.4061/2011/241652

**Published:** 2011-06-08

**Authors:** Fukuo Kondo

**Affiliations:** Department of Pathology, School of Medicine, Teikyo University, 2-11-1 Kaga, Itabashi-ku, Tokyo 173-8605, Japan

## Abstract

Stromal invasion (invasive growth of tumor tissue into portal tracts and fibrous septa) is now recognized as the most important finding in the diagnosis of the well-differentiated type of early hepatocellular carcinomas (HCCs). In differentiating stromal invasion from pseudoinvasion (benign hepatic tissue in fibrous stroma), the following 5 items are useful: (1) macroscopic or panoramic views of the histological specimen, (2) the amount of fibrous components of stroma, (3) destruction of the structure of portal tracts, (4) loss of reticulin fibers around cancer cells, and (5) cytokeratin 7 immunostaining for ductular proliferation. Knowledge of stromal invasion is also useful for a better understanding of the vasculature (hypovascular HCCs) and histological features (fatty change) of early HCCs. Invasion of preexisting arteries and portal veins causes hypo-vascularity of HCCs. Further, hypovascularity causes fatty change as a hypoxic change of cancer tissues.

## 1. Introduction

Recently, international consensus for the histological diagnosis of hepatocellular carcinoma, especially of well-differentiated type of early stage (early HCC), was published by the International Consensus Group for Hepatocellular Neoplasia (ICGHN) [1]. This was an epoch-making event for the early diagnosis and early treatment of hepatocellular carcinomas (HCCs). In this consensus paper, stromal invasion (invasive growth of tumor tissue into portal tracts and fibrous septa) was recognized as the most important finding for the diagnosis of early HCCs. Unfortunately, however, this finding is not commonly known except among a small number of liver pathology experts. To present the correct histological diagnosis of early HCCs, histological features of stromal invasion are herein explained, with details shown in many figures. It is also described how stromal invasion is closely related to characteristic image findings and histological features of early HCCs. 

## 2. History of Studies of Stromal Invasion of HCCs

Stromal invasion, formerly called interstitial invasion of HCC, is defined as invasive growth of tumor tissue into fibrous septa, portal tracts, and/or blood vessels [2–7]. Stromal invasion by other tumors of other organs is a commonly recognized concept, and has long been important evidence for the definitive diagnosis of malignant tumor [8, 9]. However, stromal invasion of HCC has not been generally known until quite recently. This finding was first reported as a “streak pattern” in the fibrous septa of cirrhosis around an HCC nodule by Kondo Y. et al. [2]. Kondo F. et al. then reported that this finding was frequently found within pre-existing portal tracts as well as fibrous septa [3], emphasizing that this finding was very useful for the diagnosis of well-differentiated HCCs. The invasion pattern was classified into 3 types—crossing type, longitudinal type, and irregular type. It was also reported that stromal invasion could be detected even by macroscopic view and by panoramic view of a histological specimen. At that time this finding was called “interstitial invasion” instead of “stromal invasion.” Tomizawa et al. reported that the growth activity of well-differentiated HCC was rather suppressed with the stromal invasion [4]. Nakano et al. divided stromal invasion into three types: (1) stromal invasion into fibrotic tissue and/or portal tracts, (2) blood vessel wall invasion of portal veins or hepatic veins, and (3) tumor thrombus [5]. Miyao et al. described that HCC tissue in the state of stromal invasion was unaccompanied by reticulin frameworks and type IV collagen [6].

In 1995, an International Working Party (IWP) of the World Congress of Gastroenterology published a consensus nomenclature and diagnostic criteria for nodular hepatocellular lesions [10]. In this article, stromal invasion was listed as a criterion for the histological diagnosis of well- and moderately differentiated HCC. Even after publication of this article, however, this finding was still not well known especially among pathologists in Western countries, possibly because related articles regarding stromal invasion were written by Japanese pathologists. This fact caused serious differences in criteria for the diagnosis of early HCCs between Eastern and Western pathologists.

In order to solve this serious problem, an International Consensus Group for Hepatocellular Neoplasia (ICGHN) was convened in April 2002 in Kurume, Japan. This group met several times and discussed histological criteria for the diagnosis of early HCCs subsequently, up to July 2007 [1]. In these meetings, the findings of stromal invasion were discussed in detail. Finally, all the participants including Western pathologists generously accepted the importance and usefulness of this finding. Park et al. reported that ductular reaction confirmed by cytokeratin 7 (CK7) is helpful in defining early stromal invasion, small hepatocellular carcinomas, and dysplastic nodules (DNs) [7]. This was the first article of stromal invasion written by a non-Japanese pathologist. All authors of this article were members of ICGHN. The authors consisted of 1 Korean, 4 Western, and 4 Japanese pathologists.

In 2009, ICGHN published the consensus paper [1], which described that stromal invasion was the most helpful in differentiating early HCC from high-grade DNs. However, this finding was not sufficiently disseminated even after publication of the consensus paper. To achieve progress in the early diagnosis of many HCC patients in the world, this finding must be explained in detail. 

## 3. How to Evaluate Stromal Invasion Correctly: Macroscopic and Histological Assessment of Stromal Invasion

**Figure 1:**
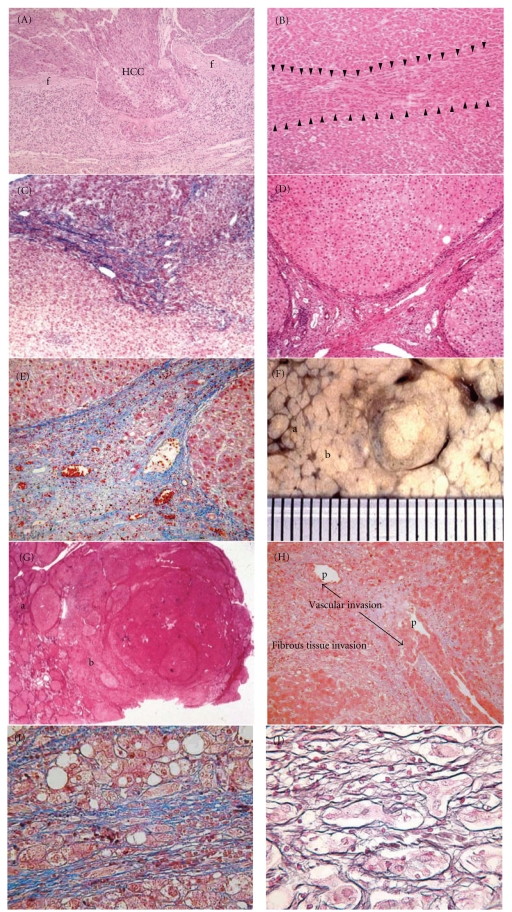

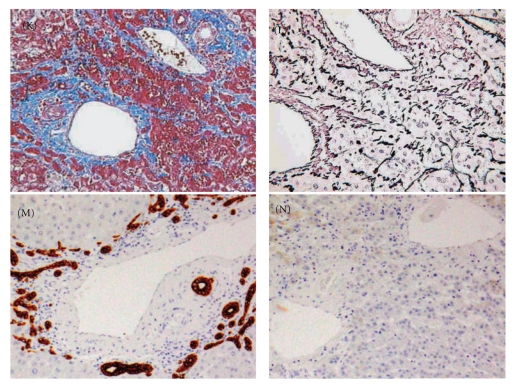
Various features of stromal invasion of hepatocellular carcinoma (HCC) and pseudo-invasion (A) Crossing type. Cancer tissue (HCC) invades across fibrous septa (f) of tumor nodule. (B) Longitudinal type. Tumor cells grow longitudinally within fibrous septa (arrowheads). (C) Irregular type. Portal areas are irregularly invaded by tumor cells (Masson trichrome stain). (D) A non-cancerous area without invasion, and a portal area and fibrous septa are clearly seen. (E) Pseudo-invasion. Benign non-cancerous cells are found in the fibrous stroma (Masson trichrome stain). (F) Macroscopic view of stromal invasion. In the non-cancerous area without invasion (area of (a)), fibrous septa are clearly seen. In the area of tumor spread (area of (b)), septa are indistinct. (G) A panoramic view of stromal invasion. In the same way as in (F), the non-cancerous area without invasion (area of (a)) shows distinct fibrous septa. The area of tumor spread (area of (b)) shows indistinct septa because stromal invasion of longitudinal type and irregular type ((B), (C)) reduced the amount of fibrous component. (H) Continuity of fibrous invasion and vascular invasion. The arrows show portal vein (p) invasion. Vascular invasion is continuous to stromal invasion of fibrous tissue of the portal “tract” and fibrous septum (Masson trichrome stain). (I) Masson trichrome staining of pseudoinvasion. (J) Silver staining of the same specimen as (I). Liver cells are clearly surrounded by reticulin fibers. (K) Masson trichrome staining of true invasion. (L) Silver staining of the same specimen as (K). Carcinoma cells are not surrounded by reticulin fibers. (M) (N) Cytokeratin (CK) 7 immunostaining in a non-cancerous area (M) and cancerous area (N). (M) Ductular reaction, confirmed by CK 7 staining, is clearly seen in a non-cancerous, non-invasive area. (N) Ductular reaction is not found in the invasive area. (N) Adapted from Y. Kondo et al. [2], F. Kondo et al. [3], and from F. Kondo [11].

 Stromal invasion is invasive growth of tumor tissue into stroma (fibrous septa, portal tracts, and/or blood vessels). It is histologically classified into 3 types—crossing type, longitudinal type, and irregular type (Figures 1(A), 1(B), and 1(C) [4].

In the crossing type, HCC invades across fibrous septa of tumor nodules (Figure 1(A)). In the longitudinal type, tumor cells grow longitudinally within fibrous septa (Figure 1(B)). In the irregular type, portal areas are irregularly invaded by tumor cells (Figure 1(C)). The crossing type is usually observed in moderately or poorly differentiated HCCs whereas the longitudinal and irregular types are usually found in well-differentiated HCCs, although also at times in moderately or poorly differentiated HCCs. In the evaluation of stromal invasion, comparison of cancer areas with noncancerous areas is very useful (Figure 1(D)), and we have to differentiate “pseudo-invasion” from true stromal invasion. Pseudo-invasion means benign non-cancerous tissue in the fibrous stroma (Figure 1(E)), and this does resemble stromal invasion. 

 For the differentiation, the following factors are very useful.

1. Macroscopic and/or panoramic (low-magnification) views of the nodule.
2. Amount of fibrous components of the stroma.
3. Continuity to vascular invasion and destruction of the structure of portal tracts.
4. Loss of reticulin fibers around tumor cells.
5. Cytokeratin 7 immunostaining.

Stromal invasion can be identified even by a macroscopic and/or panoramic view of histological specimens. As is seen in Figure 1(F) (macroscopic view of HCC), in the non-cancerous area without invasion (area of (a)), the fibrous septa are clearly visible. However, in the area of tumor spread (area of (b)), the septa are indistinct. Similarly, in a panoramic view of a histological specimen of HCC (Figure 1(G)), distinct fibrous septa (area of (a)) and indistinct fibrous septa (area of (b)) can be clearly identified. In these indistinct septa, tumor invasion was then detected by microscope (Figures 1(B) and 1(C)). The amount of the fibrous component is quite different between the invasive and noninvasive areas, an important point for the differentiation from pseudo-invasion. The amount of the fibrous component was decreased as a result of the tumor invasion, and this decrease caused the indistinctness of the fibrous septa. Pseudo-invasion is usually caused by fibrosis around benign non-cancerous liver tissue. Therefore, it does not show reduction in the fibrous component. When stromal invasion is very mild and fibrous components are minimally reduced, histological and macroscopic assessment of stromal invasion is difficult. However, stromal invasion is severe enough, histological and macroscopic assessment is easy (Figures 1(F) and 1(G)). Even in cases of HCCs with minimal invasion and DNs, proportion of fibrous stroma or portal tracts are reduced to some extent as described later. Therefore, macroscopic view is helpful for recognizing eHCC and DN. The continuity to vascular invasion and destruction of the structure of portal tracts are also important findings (Figure 1(H)). The former is a decisive finding of malignancy. Although it is not a common finding, it can be detected in some early HCCs. Tumor tissue first invades into fibrous septa, then into vascular walls, and finally into vascular lumina. The connection among endothelial cells was most certainly destroyed by the mechanical force exerted by tumor growth. Portal vein invasion in Figure 1(H) is in vascular space. It means tumor cells are disseminated in circulation. However, tumor cell dissemination does not directly cause metastasis. Before forming metastatic foci, tumor cells have to survive within circulation, have to reach to remote areas, have to invade vascular walls from inside to outside, and have to proliferate outside the blood vessels. Interpretation of tumor cells in the subendothelial space is controversial. It can be true sub-endothelial invasion. However, it can be interpreted as blood space invasion after re-covering with endothelial cells. Endothelial cells can easily cover intravascular foreign substance. Destruction of the portal tract structure is more frequently found in stromal invasion while this feature is not seen in pseudo-invasion (Figure 1(E)).

Loss of reticulin fibers around the tumor cells is another useful finding [7]. Figures 1(I) and 1(J) show Masson trichrome staining and silver staining of pseudo-invasion. And Figures 1(K) and 1(L) show those of true invasion, respectively. Magnification of Figures 1(J) and 1(L) is a little higher than that of Figures 1(I) and 1(K). The liver parenchyma is clearly surrounded by reticulin fibers in the pseudo-invasion (Figure 1(J)). By contrast, the liver tissue of the true invasion lacks such surrounding reticulin fibers (Figure 1(L)). Tumor cells are embedded in the septal fibers without being clothed by reticulin fibers.

As described above, Park et al. reported that CK7 immunostaining is useful for identifying stromal invasion [7]. Ductular reaction confirmed by CK7 staining is frequently found in non-cancerous hepatocellular nodular lesions (Figure 1(M)) while it is less frequently found in HCCs with true stromal invasion (Figure 1(N)). Ductules around the fibrous septa are non-cancerous components. They must have been invaded by well-differentiated HCC cells around the fibrous septa or by HCC cells from the fibrous septa.

For the correct assessment of true stromal invasion, these silver and CK7 stainings are useful. Masson trichrome stain, Azan-Mallory stain, and Victoria blue stain are also useful for clarifying the fibrous components.

**Figure 2:**
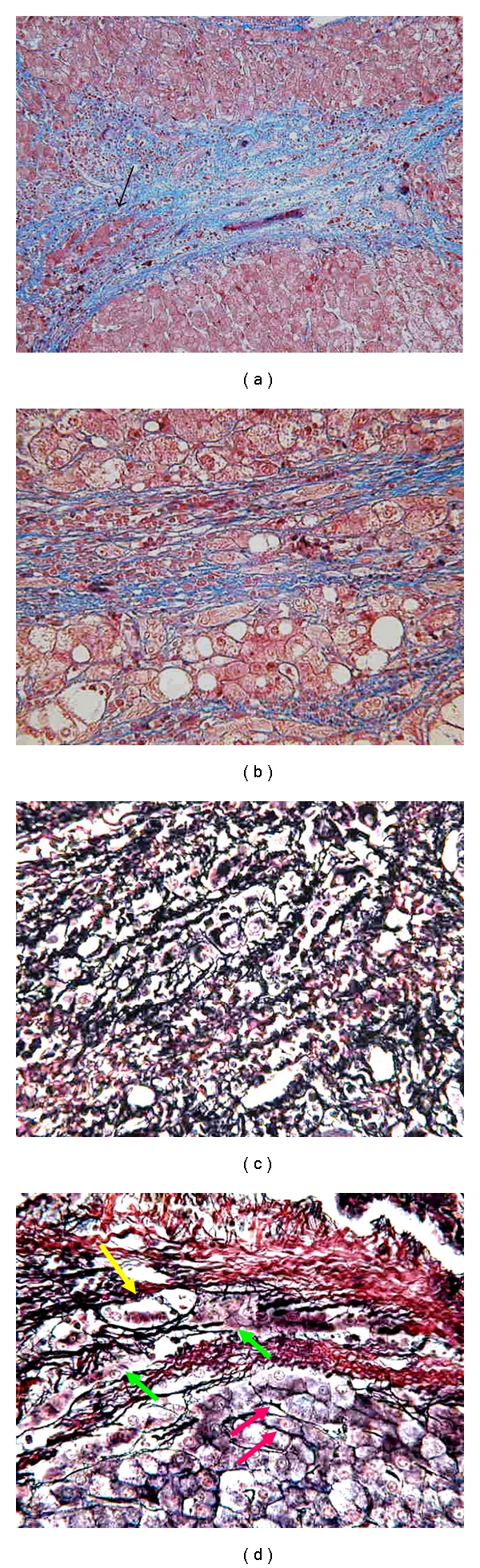
Histological features which make the assessment of stromal invasion difficult (a) True stromal invasion of very mild grade. The fibrous septum is almost intact except for a small area (arrow). (b) Pseudo-invasion consisting of very thin fibrous bundles within and around thick liver cell cords. This pattern was formed by dissection of liver parenchyma by very thin fibrous tissue. (c) A specimen of very poorly performed silver stain. (d) Silver stain of HCC tissue within and around a fibrous septum. Reticulin fibers circumscribing cancer tissue are seen even in the area of true invasion (yellow arrow). However, noncircumscribed tumor cells are also seen in the same fibrous septum (green arrows). This area is a “battle front” of invasion. Red arrows show ordinary tumor tissue with reticulin fibers surrounding the fibrous tissue.

Next, some histological features that make the assessment of stromal invasion difficult must be shown (Figure 2). 

Figure 2(a) shows true stromal invasion of a very mild grade. The fibrous septum is almost intact except for a small area (arrow). By contrast, Figure 2(b) shows pseudo-invasion consisting of very thin fibrous bundles within and around thick liver cell cords. This pattern was not formed by the reduction of fibrous component but rather by dissection of liver parenchyma by very thin fibrous tissue. Observing these two figures, pathologists may doubt the concept of stromal invasion. In such cases, however, silver stain is very useful. Reticulin fibers are lost in the case of true invasion but not in the case of pseudo-invasion. In cases like Figure 2(a), I recommend pathologists to search for more severely invaded portal tracts that can easily be assessed as true invasion. Even very well-differentiated HCCs sometimes include severely invaded portal tracts as well as minimally invaded portal tracts.  Figure 2(c) shows a specimen of very poorly performed silver staining. Such poorly stained specimen makes the diagnosis difficult.

Figure 2(d) shows that reticulin fibers sometimes circumscribe cancer tissue even in the area of true invasion (yellow arrow). However, noncircumscribed cells are usually seen in the same fibrous septum (green arrows). This area is a “battle front” of invasion. By contrast, red arrows show ordinary tumor tissue with reticulin fibers outside the fibrous tissue. In fact, reticulin fibers are sometimes observed within and around true invasive areas. After the invasive process is over, the cancer cells form ordinary cancer areas. In such phase of tumor growth, reticulin fibers must be formed again. 

## 4. Influence of Stromal Invasion on Images and Histological Features of Early HCCs

**Figure 3:**
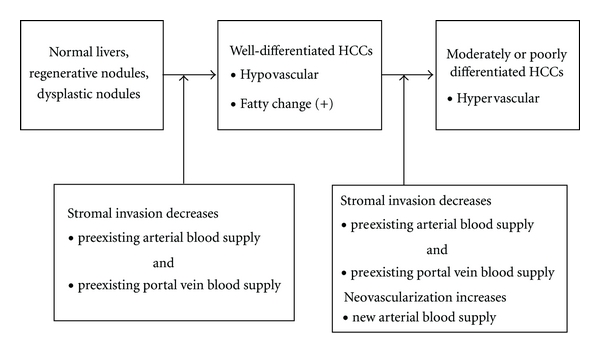
Relationship between cancer development, vascularity, histological feature (fatty change), and stromal invasion.

Stromal invasion is closely related to the images and histological features of early HCCs.  Figure 3 shows the relationship between cancer development, vascularity, histological feature (fatty change), and stromal invasion. Although there exists no direct evidence in a strict meaning, the possibility or hypothesis shown in Figure 3 well explains the formation mechanism of vascularities and fatty change of HCC. At least, previous studies [12–18] can be good indirect evidence for the description in Figure 3. Well-differentiated HCCs emerge from non-cancerous liver tissues (normal liver, regenerative nodules, and DNs), and then they progress into moderately or poorly differentiated HCCs. Vascularity (usually evaluated by contrast medium-enhanced images) changes during this process. Well-differentiated HCCs are usually hypo-vascular lesions [12–17]. This hypo-vascularity means a decrease in pre-existing arterial and portal venous blood supply caused by stromal invasion. During the process in which a well-differentiated HCC progresses into a moderately or poorly differentiated HCC, vascularity usually changes to become hypervascular [12–17]. This vascular change is caused by proliferation of abnormal arteries (neovascularization) [1, 17].

As a matter of fact, abnormal arteries are found within DNs [1, 17]. However, the increase of arteries is not sufficient to cause hyper-vascularity. DNs sometimes show hypo-vascularity without stromal invasion [17]. This hypo-vascularity is attributed to a relative decrease of density of pre-existing portal tracts. Because the parenchymal component increases within the DN nodule, the density of pre-existing portal tracts decreases. After DNs transforms into early HCCs, the density may decrease more severely by stromal invasion. This process must have caused hypo-vascularity of early HCCs.

This hypo-vascularity can also explain the formation mechanism of fatty change, a well-known feature of early HCCs (Figure 3) [18]. Although fatty change may be attributed to metabolic change of tumor cells with tumor development independent of hypoxic change, hypo-vascularity may cause fatty change as a hypoxic change. 

As mentioned above, knowledge of stromal invasion is very useful to understanding the vascularity and histological features of early HCCs. 

## 5. Limitations of Assessment of Stromal Invasion

Finally, limitations of the assessment of stromal invasion have to be described. Stromal invasion cannot always be assessed histologically, and it is very rarely assessed in the examination of thin-needle biopsy specimens [11]. Biopsy specimens are simply too small to allow examination of stromal invasion. For this reason, very well differentiated HCCs lacking typical features of ordinary well-differentiated HCCs are not diagnosed by biopsy [11]. As histological criteria for the biopsy diagnosis of well-differentiated HCCs, (1) nuclear crowding (hypercellularity), (2) hyperstainability of cytoplasm (basophilia or eosinophilia), and (3) microacinar formation have been used til now [19]. These criteria have been proved to be useful because ordinary well-differentiated HCCs have considerable parenchymal atypia. However, some very well-differentiated HCCs are not diagnosed by biopsy and are definitively diagnosed after examination of stromal invasion in resected specimens.

To make progress in the early diagnosis of HCCs, we have to develop new parenchymal tumor markers that can be used for biopsy diagnosis. Some attempts have been made recently to utilize immunohistochemical markers for the diagnosis of well-differentiated HCCs [20–25]. Heat shock protein 70 (HSP70) [20, 21], glypican 3 (GPC3) [20, 22, 23], and glutamine synthetase (GS) [20, 24, 25] have been used independently or in combination. At present, these markers are used in a complementary manner to morphological criteria. Newer markers have also been tried [26, 27]. We are hopeful that excellent markers with high sensitivity and high specificity are developed in the future. 

## 6. Conclusions

1. Stromal invasion is a very important finding for the histological diagnosis of early HCCs.
2. For the correct assessment of stromal invasion, the following 5 items are useful: (1) macroscopic or panoramic views of the histological specimen, (2) amount of fibrous components of the stroma, (3) destruction of the structure of portal tracts, (4) loss of reticulin fibers around cancer cells, and (5) CK 7 immunostaining for ductular proliferation.
3. Knowledge of stromal invasion is very useful to understand the formation mechanism of images (vascularity) and histological features (fatty change) of early HCCs.
4. Stromal invasion cannot be assessed in thin-needle biopsy specimens.
5. New parenchymal tumor markers usable for biopsy diagnosis need to be developed.
